# Assisting clinical diagnosis with interpretable fuzzy probabilistic modelling

**DOI:** 10.1186/s12911-025-03183-5

**Published:** 2025-09-15

**Authors:** Giulia Capitoli, Marco S. Nobile, Emma L. Ambags, Vincenzo L’Imperio, Michele Provenzano, Pietro Liò

**Affiliations:** 1 School of Medicine and Surgery, University of Milano-Bicocca, 20854 Monza, Italy; 2 Biostatistics and Clinical Epidemiology, Fondazione IRCCS San Gerardo dei Tintori, 20854 Monza, Italy; 3 Bicocca Bioinformatics, Biostatistics and Bioimaging (B4) Research Center, 20854 Monza, Italy; 4 Department of Industrial Engineering & Innovation Sciences, Eindhoven University of Technology, Eindhoven, 5612 AZ The Netherlands; 5 Department of Environmental Sciences, Informatics and Statistics, Ca’ Foscari University of Venice, 30170 Venice, Italy; 6 Department of Medicine and Surgery, Pathology, University of Milano-Bicocca, IRCCS Fondazione San Gerardo dei Tintori, 20900 Monza, Italy; 7 Department of Pharmacy, Health and Nutritional Sciences, University of Calabria, 87036 Arcavacata di Rende, Italy; 8 Nephrology, Dialysis and Transplant Unit, ”SS. Annunziata” Hospital, 87100 Cosenza, Italy; 9 Department of Computer Science and Technology, University of Cambridge, Cambridge, CB3 0FD UK

**Keywords:** Interpretable AI, Clinical decision support, Fuzzy logic, Probabilistic decision trees, Thyroid cancer, Chronic kidney disease

## Abstract

**Background:**

The need for transparency and interpretability is a fundamental theme to be addressed by Artificial Intelligence (AI) research, especially in high-risk applications such as healthcare. In this work, we propose Fuzzy Sets in Probability Trees (FPT), a novel method that combines probabilistic trees and fuzzy logic. This approach is fully interpretable, providing clinicians with a tool generate and verify the entire clinical decision process.

**Methods:**

FPT extends the existing framework of Probabilistic Decision Trees by incorporating the uncertainty in the data, allowing for a flexible description of vague variables. Thus, FPTs enable the incorporation of domain knowledge in the form of fuzzy membership functions within the framework of probabilistic trees. Furthermore, FPTs can represent circumstances or explanations that cannot be represented with other techniques (e.g., Bayesian networks), paving the way to a novel form of interpretable AI that allows clinicians to generate, control and verify the entire diagnosis procedure; one of the strengths of our methodology is the capability to decrease the frequency of misdiagnoses by providing an estimate of uncertainties and counterfactuals.

**Results:**

We applied FPT to two real medical scenarios: classifying malignant thyroid nodules, and predicting the risk of progression in chronic kidney disease patients. Our results show that FPTs can provide interpretable support to clinicians. We also show that FPT and its predictions can assist clinical practice in an intuitive manner, with the use of a user-friendly interface specifically designed for this purpose.

**Conclusion:**

The integration of probabilistic trees and fuzzy reasoning preserves the nuances that are generally lost in (probabilistic) decision trees due to the adoption of crisp thresholds, leading to hybrid trees that provide an AI system better aligned with human reasoning processes and that can effectively support clinicians in the diagnosis decision process.

**Graphical Abstract:**

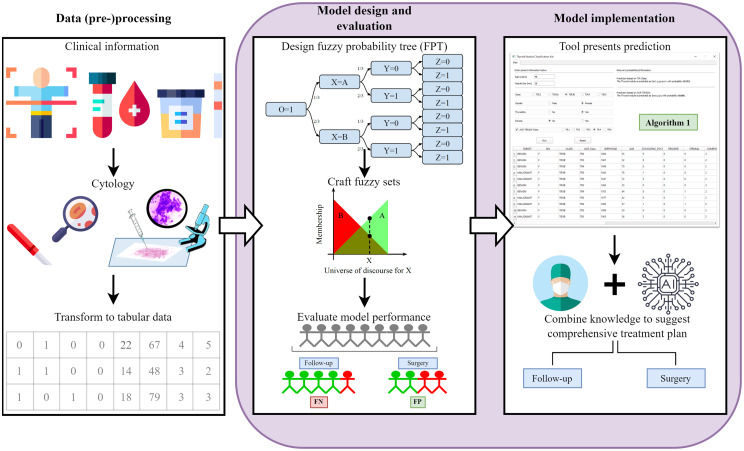

**Supplementary Information:**

The online version contains supplementary material available at 10.1186/s12911-025-03183-5.

## Background

With the extraordinary growth of biomedical data, the need for computational methods to assist medical practice is naturally emerging. A Clinical Decision Support System (CDSS) is an information system designed to assists the decision making of healthcare providers, in order to improve patient care. These systems provide assistance during complex decision making by offering targeted clinical knowledge, care plan recommendations, and other relevant health information at the point of care. AI systems – trained for a specific task [1] – working for human experts have been found to provide better accuracy and efficiency [2]. Occasionally, these systems can even outperform human experts, are faster, and not prone to fatigue and stress. As a result, patient care can be improved and, simultaneously, practice variability can be reduced.

Regardless of the success of Machine Learning (ML) and Artificial Intelligence in several disciplines, the adoption of such approaches to assist decisions in high-risk domains is still limited due to trust and transparency issues [3]. In the clinical field, black box methods are unacceptable [4] because the healthcare provider must be able to justify the rationale behind any decision, and the patient has the right to receive a meaningful explanation. The black box problem is a well known shortfall of both AI and ML, where a user enters an input and receives a response from the system but cannot get a rationale for the output. Therefore, it is important that the rationale of predictions and recommendations that are produced by a CDSS are understandable and interpretable to users, maximizing the trust and acceptance of the generated suggestions.

The need for transparency and interpretability is increasingly being recognized as a central theme to be addressed by AI research, especially in the case of healthcare [5, 6]. In order to prevent unfair treatment on a large scale, regulations and international treaties have been introduced. For example, the General Data Protection Regulation (GDPR) [7] and the Artificial Intelligence Act (AIA) [8], both proposed by the European Union, strive for a positive and acceptable use of models and intelligent methods. These regulations are designed to shield citizens from decision making purely based on black boxes and mandate a right to an explanation. The proposed rules are specifically aimed at regulating the development and use of AI. These proposed regulations impose AI systems to comply with a set of mandatory requirements for trustworthy AI to be allowed to be placed on the EU market, primarily to enforce transparency and human oversight obligations to high-risk AI systems, e.g., safety-critical systems such as systems deployed in clinical environments (see also [9]). CDSSs specifically, as they operate in a sensitive field, are allowed but remain subject to the strictest set of requirements [8]. Therefore, in order to effectively support the decision making process, computational methods need to be explainable and interpretable [10] and there must be an interplay between human operators and AI.

The potential of CDSSs is evident and the need for these systems to be interpretable is ever growing, leading to hundreds of works exploring different approaches [11]. In practice, end-users (i.e., healthcare providers) of CDSSs are less likely to trust the recommendations of systems whose workings they do not understand [12]. Moreover, CDSSs will be challenged legally by having to comply with future requirements as imposed by the AIA. Therefore, this research focuses on Interpretable AI (IAI) rather than eXplainable AI (XAI).

XAI methods use *post hoc* analysis of the decision making process, so that it provides insights in the way decisions have been taken by the model. Thus, XAI only peers inside the model after it has been created. Concretely, an XAI model will first involve building a black box model, after which it will help to dissect the internal mechanics of the black box model to understand the importance of various features and the decisions it can lead to [13]. IAI creates models that are *a priori* interpretable by humans, i.e., human-interpretable from the beginning [14]. The decision-making process of the system is directly observable. Interpretability is considered a broader term than explainability [13]. An interpretable system is where the user can not only see but also understand how inputs are mathematically mapped to outputs [15].

This paper presents an interpretable AI method that integrates Fuzzy Logic (FL) and Probabilistic Trees (PTs), extending the work by Genewein et al. [16]. PTs have highly self-explanatory semantics, being able to accurately represent and structure a decision problem [17]. The presentation of a PT is descriptive and simple to understand, and intuitively visualizes conditional probabilities that build on Bayes’ theorem [18]. In this context, the importance of evaluating the uncertainty and vagueness in medical variables should be considered. Think of ill-defined concepts such as: ‘young’ vs ‘old’, ‘small’ vs ‘large’, or a concept such as having a ‘high fever’ at a body temperature of $39^{\circ}$C (but what about a temperature of $38.9^{\circ}$C?). The theory of fuzzy sets, introduced by Zadeh [19], gives the possibility to formalize a partial membership of an element to a fuzzy set, so that any (clinical) variable can have multiple characterizations at once, to differing degrees. This concept allows for incorporating fuzzy relationships, and thus extending the possibilities of PTs.

**Fig. 1:**
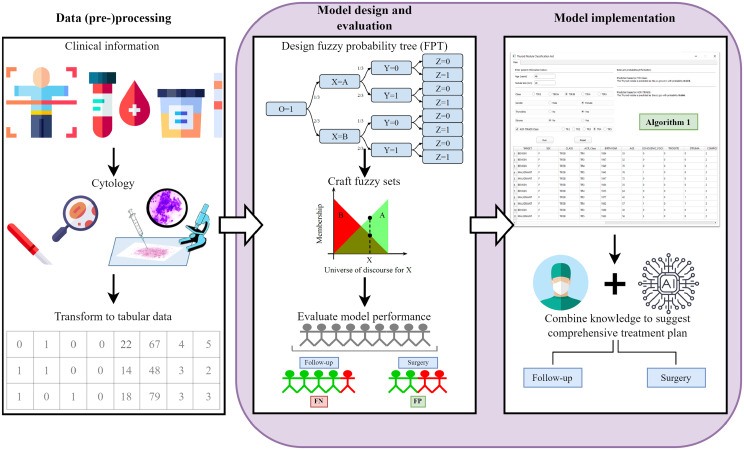
Visual abstract of the approach taken within this research; modeling and decision support for clinical decision making. The first phase occurred outside this research, the data has been provided in tabular form. The phases in the purple rectangle have been carried out within this research. Firstly, the modeling phase is carried out (creation of the tree, crafting fuzzy sets, and assessing the performance). Secondly, a tool has been created to assist clinical decision making in an interpretable manner. The green label “algorithm 1” indicates where in the decision making process the fuzzy probabilistic prediction algorithm acts

In this work, we propose a novel approach based on Probabilistic Trees integrating fuzzy sets (named Fuzzy Probabilistic Trees, or FPT) and we apply the method to assist in decision making in two clinical contexts, namely: (1) the classification of thyroid nodules, and, (2) determining the risk of patients progressing to the final and most harmful stage of chronic kidney disease over a 2-year period. Two different types of diseases are used for the case studies in order to test out and substantiate the generalizability of the FPTs across differing diseases. Figure 1 visualizes the steps involved in each case study. The phases highlighted by the purple rectangle are executed within this research. The green label containing “Algorithm 1” in the column on the right indicates where the fuzzy probabilistic decision tree is used in the decision making process.

The integration of probabilistic trees and fuzzy reasoning, leading to a hybrid tree, will provide an AI system that is aligned with the way humans reason. The performance of the fuzzy probability tree is compared to several other interpretable decision making models, namely regular PTs, Decision Trees (DT), and Logistic Regression (LR). In order to promote usability and interpretability, we created a tool that provides an interactive and natural way to interact with the decision making algorithm. The tool can be used to identify records of past patients with similar profiles based on the fuzzy probabilistic tree.

This manuscript is organized as follows. Existing methods on which this paper builds are outlined in Sect. 2. Section 3 describes the proposed FPT decision making method. The two case studies used to assess the performance of FPT are described in Sect. 4, this section also outlines the creation of the trees and the fuzzy sets, as well as providing a walk-through of an example showing how the algorithm makes predictions. Section 5 presents the achieved experimental results of the FPT compared to other interpretable benchmark models. Finally, discussions and conclusive remarks are presented in Sects. 6 and 7, respectively.

## Methods

### Probabilistic trees

**Fig. 2:**
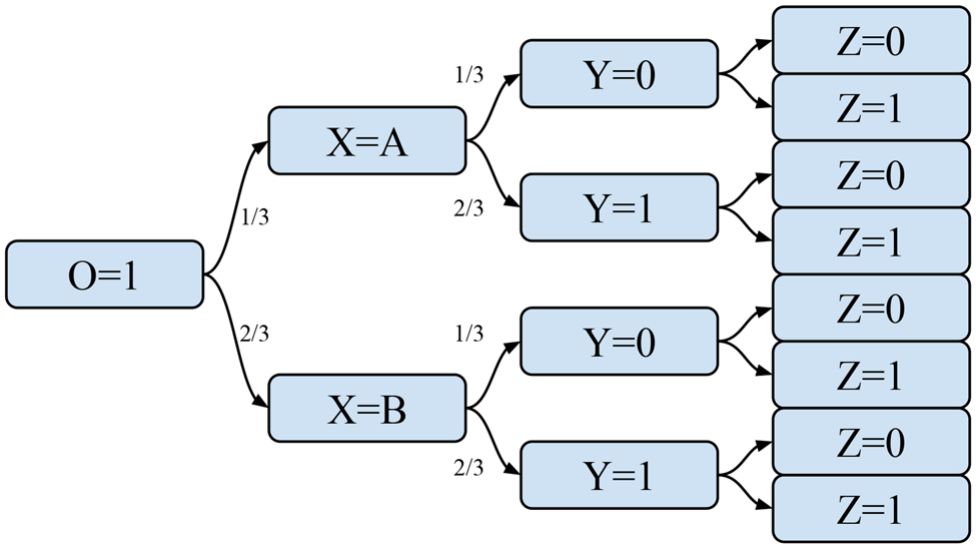
Scheme of a probabilistic tree with 3 variables

(Discrete) Probability Trees are causal models that use nodes to represent the potential states of the processes, and the arcs between nodes indicate both the probabilistic transitions and the causal dependencies between them [16]. Similarly to Bayesian Networks (BNs), they can be used to model causal relationships and perform inference. However, thanks to the fact that they are not represented as a directed acyclic graph, PTs allow to model multiple alternative scenarios where variables do not necessarily follow a partial order. Formally, the nodes modelling the variables in a PT are tuples $n=(w, \mathcal{S}, \mathcal{C})$ where: $w \in \mathbb{N}$ is a numeric identifier for the node within the tree; $\mathcal{S}$ is a list of statements and $\mathcal{C}$ is a ordered set of transitions $(\theta, m) \in [0, 1] \times \mathbb{N}$ where $\theta$ is the transition probability to the child node $m$. Every transition probability in the PT is the result of a conditional probability to go from one node ($w$) to the next ($m$): $P(S_m | S_w) = \theta_{w,m}$, where $S_m$ and $S_w$ are the statements associated to nodes $m$ and $w$, respectively. A statement has the form ‘$X$ IS something’, which is technically represented as a tuple ($X$, something) $\in \chi \times \mathcal{V}_\chi$ where $\chi$ is the set of variables and $\mathcal{V}_\chi = \{V_X\}_{X \in \chi}$, specifically $\mathcal{V}_X$ is the range of possible values of variable $X$. Such range is sometimes well defined (e.g., concepts like ‘p53 mutation = True’), but this is often not the case in real world scenarios where variables are more fuzzy (e.g., concepts like ‘fever = High’). Of course, such fuzzy concepts can, in principle, be converted to crisp concepts by using thresholds. This is a rather used approach (e.g., ‘high fever’ corresponds to a temperature $\geq 38^{\circ}$C) that unfortunately can introduce artifacts, it defines arbitrary partitions, and in general prevents us from properly handling uncertainty in our data (e.g., what about a temperature of $37.9^{\circ}$C?). A (*total*) *realization* in the probability tree is a path from the root to a leaf, and its probability is obtained by multiplying the transition probabilities along the path. An *event* is a collection of total realizations that we can filter using propositions about a random variable. Therefore, an event is used to describe a set of all realizations that contain a node with the specific statement, for instance the event ‘$X=A$’ in Fig. 2. Furthermore, we can use logical connectives of negation (Not, $\neg$), conjunction (And, $\land$) and disjunction (Or, $\lor$) [16]. With this we can state composite events, such as ‘$\neg (X = A \land Y = 1)$’.

### Fuzzy sets

Human reasoning is able to process information expressed in vague and uncertain terms. The theory of fuzzy sets, introduced by [19], gives the possibility of formalizing a partial membership of an element (of a universe of discourse) to a fuzzy set. Specifically, the membership degree $\mu(x)$ of an element $x$ can range from 0 ( = no membership at all) to 1 ( = full membership). We can mathematically define a fuzzy set $\widetilde{A}$ as:

1 $$ \widetilde{A} = \{(u, \mu_{\widetilde{A}}(u)) \mid u \in U\}$$

Here, $\mu_{\widetilde{A}}(u)$ is the degree of membership of $u$ in the fuzzy set $\widetilde{A}$, assuming $\mu_{\widetilde{A}} (u) \in [0, 1]$.

Fuzzy sets can be used to express linguistic terms (e.g., “low”, “high”) that, in turn, can be used to express linguistic variables, i.e., variables whose values are concepts instead of crisp numbers. Thanks to this approach, fuzzy sets can represent concepts in a way that is as close as possible to human natural reasoning. Thanks to fuzziness, we can also express concepts that are vague, uncertain, or ill-defined. This possibility is here exploited to express medical concepts that are intrinsically fuzzy in nature (e.g., a young vs an elderly person). We provide some examples of fuzzy sets applied to biomedical terminology in Fig. 3 of Supplementary Material.

Fuzzy sets have gradual boundaries, in contrast to classical sets which are based on discrete boundaries. The universe of discourse ($U$) is the set of possible values that a variable *u* can take on.

## The proposed FPT

We propose the integration of PTs and Fuzzy set theory to build Fuzzy Probability Tree, to accomodate uncertainty and vagueness for a subset of variables. This allows for using the existing framework of PTs as developed in [16], while incorporating uncertainty about the data, or allowing for a flexible description of vague variables. Thus, enabling us to incorporate human expert knowledge in the form of fuzzy membership functions to probabilistic trees.

While PTs require well defined discrete concepts and events, this is seldom the case in real world scenarios. Especially in the medical field, where the variables are often fuzzy, vague or ambiguous. By using the FPT approach – and by means of carefully crafted fuzzy sets – we can incorporate the inherent uncertainty in variables to balance the probabilities. The integration of PTs and fuzzy sets, leading to an FPT, will provide an AI method that is aligned with the way humans reason. Furthermore, (F)PTs are able to represent circumstances or *explanations* that cannot be represented with other graphical techniques (e.g., Bayesian networks), paving the way to a novel form of interpretable AI.

Constructing the FPT is an iterative process, done in collaboration with domain experts. The selection of the features and the order of the features in the tree are based on domain knowledge (deduction), however, the transition probabilities in the trees are based on the data (induction). In making its predictions, the tree will present two probabilities derived from the FPT that has been constructed. It will present the probability associated with the data point belonging to the negative class (0: no disease) or positive class (1: disease), naturally these probabilities sum to 1. By default we consider a cut-off at a probability of 0.50, which indicates that when the probability of the positive class is $\geq 0.50$ the model predicts the nodule as the positive class. By this we can transform the probabilities into binary predictions, which can be used to determine the overall performance of the probabilistic model. Although, part of the strength of the model lies in its ability to show the two probabilities, and with it provide its degree of certainty that the nodule is either predicted as negative or positive. This threshold can be modified to reflect the situation at hand. For example in times where prioritisation is required, the threshold can be increased to consider the more urgent/certain cases.

Algorithm 1 takes as input the FPT, a pointer to a specific node of the FPT, a list of statements, and a specific class. The idea is to transverse the tree, characterise a specific patient with respect to the specified statements, and calculate the probability of the patient to belong to the given class.

**Figure:**
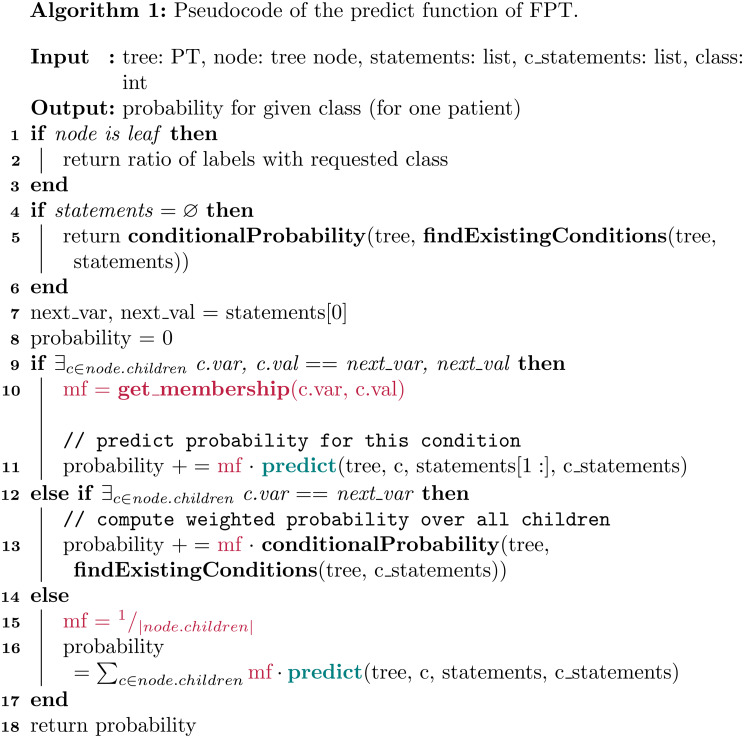

The algorithm begins by considering the base case of tree leaf (lines 1–3). In such a case, we simply count and return the number of cases for the requested class and terminate the execution. In lines 4–6, the algorithm checks whether the list of statements is empty. In this case, the algorithm uses the conditionalProbability helper function which calculates the probability of a specific class (e.g., malignancy) occurring given a set of conditions. Else, the algorithm extracts the next statement (line 7), in the form of pair (variable,value), and checks whether there exists a child node corresponding to that specific statement. If so, the fuzzy membership corresponding to this case is calculated (if the variable is not fuzzy, it is set to 1 by default). The membership is then multiplied by the probability of the rest of the sub-tree by means of a recursive call (lines 10–11). If the pair in the statement was not found, the algorithm still tries to determine whether there is a child node corresponding to the variable. In this second case, it calculates the weighted probability across all children (lines 12–13). Finally, if the variable does not exist as child, the algorithm calculates the probability of the chosen event by following all children, and weighs each prediction using a membership value equal to 1 divided by the number of children nodes (lines 15–16).

The algorithm exploits the helper function Find Existing Conditions (see Algorithm 2), which is called whenever a patient in the test set is not represented in the training set, that is, there is not a path in the tree that fully represents that patient. Then we wish to predict this patient using the weighted average of similar data points. In order to do so, this function returns a list of the largest set of conditions of this patient that is represented in the tree. In essence, the function finds the longest subset of statements that exist in the tree, which is crucial as it enables the model to generalize beyond exact matches and utilize partial similarities within the data. By selecting the most comprehensive set of existing conditions, the algorithm ensures that the prediction is based on the most relevant patterns observed in the training set. The likelihood of encountering test instances that do not have an exact match in the tree decreases as the size of the dataset increases relative to the complexity of the decision tree. As the dataset grows, it is expected to encompass a wider range of possible combinations of conditions, thereby reducing the chances of encountering entirely novel instances and improving the model’s coverage of the underlying data distribution.

**Figure:**
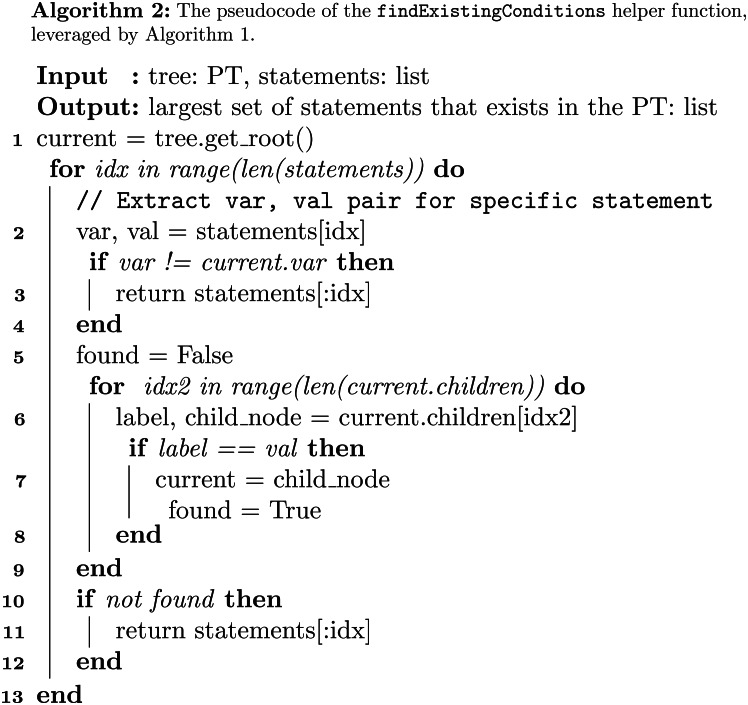

## Two clinical case studies

In this section, the proposed FPT method will be applied for predictions in two real-world medical scenarios. In the first use case, the FPT will be used for the classification of malignant and benign thyroid nodules. We will provide detailed descriptions about the tree building process, the inner workings of the tree, and we will elaborate on the differences between classifying using regular PTs and the FPT method. In both examples, the features were selected manually, with the assistance of domain experts.

Thereafter, we will discuss the case of predicting the 2-year risk of patients suffering from Chronic Kidney Disease (CKD) to progress to the most advanced stage of this disease, namely the End Stage Renal Disease (ESRD) where patients typically undergo dialysis and experience kidney failure. This second case study serves to substantiate the generalisability of our approach.

We think it is interesting to present two completely unrelated case studies, based on two different types of diseases and datasets, so that the performance of the FPT can be examined carefully. The two diseases are different in terms of the classifications introduced by the International Statistical Classification of Diseases and Related Health Problems (ICD). Thyroid cancer falls under the category of neoplasms (ICD-10 Code: C73) and CKD belongs to the diseases of the genitourinary system (ICD-10 Code: N18) [20].

### A thyroid nodule case study

Thyroid nodules are lumps that form within the thyroid gland. They are extremely common and a frequent find during neck sonography exams: as a matter of fact, approximately half of the patients have at least one thyroid nodule by the age of 60 [21]. However, only 5 to 10% of thyroid nodules are found to be cancerous [22–24]. How to distinguish between benign and malignant thyroid nodules is a great clinical challenge, one in need of solving in order to perform the appropriate surgery with the correct indication. Patients that undergo surgery to remove (part of) the thyroid gland, run the risk of needing lifelong hormone replacement therapy. However, many patients still undergo an unnecessary surgery in response to their thyroid nodules, which is burdensome for the patient and expensive for the healthcare providers. Since the risks are high in the clinical field of predicting thyroid nodules, an effective yet interpretable model to assist in the complex decision making process of clinicians is considered very valuable.

#### The thyroid nodule data set

This study includes real medical case data, including 448 patients who underwent Fine Needle Aspiration (FNA), guided by the United States Thyroid Imaging Reporting and Data Systems (TIRADS). The patients were under treatment at the interventional radiology clinic, ASST Monza, Italy between the months of January and August of 2019 [25, 26]. The original data set is of tabular form and contains the information on a total of 480 thyroid nodules that were subjected to FNA, each record represents a thyroid nodule case in a specific patient containing the relevant information for 33 features. Table 1 presents an overview of the relevant features and their types. From the 480 thyroid nodules, a total of 79 were excluded: 13 lesions with an unsatisfactory cytology and no FNA repetition; and 66 nodules with no histopathology/follow-up diagnosis available. Resulting in a total of 401 subjects to be considered in developing the model. Appropriate informed consent have been obtained from all patients.

**Table 1:** Explanation of the features of the thyroid dataset

Description	Type
Date of birth	Ordinal
Gender	Binary
Date of FNA	Ordinal
Thyroid suspiciousness class	Ordinal
Final classification	Ordered
Thyroiditis indication	Binary
Struma indication	Binary
Composition of thyroid nodule	Categorical
Echogenicity of thyroid nodule	Categorical
Shape of thyroid nodule	Categorical
Margins of thyroid nodule	Categorical
Measure presence of echogenic foci	Categorical
EU TIRADS indication	Categorical
ACR TIRADS indication	Categorical
Thyroid nodule dimensions	Quantitative

**Fig. 3:**
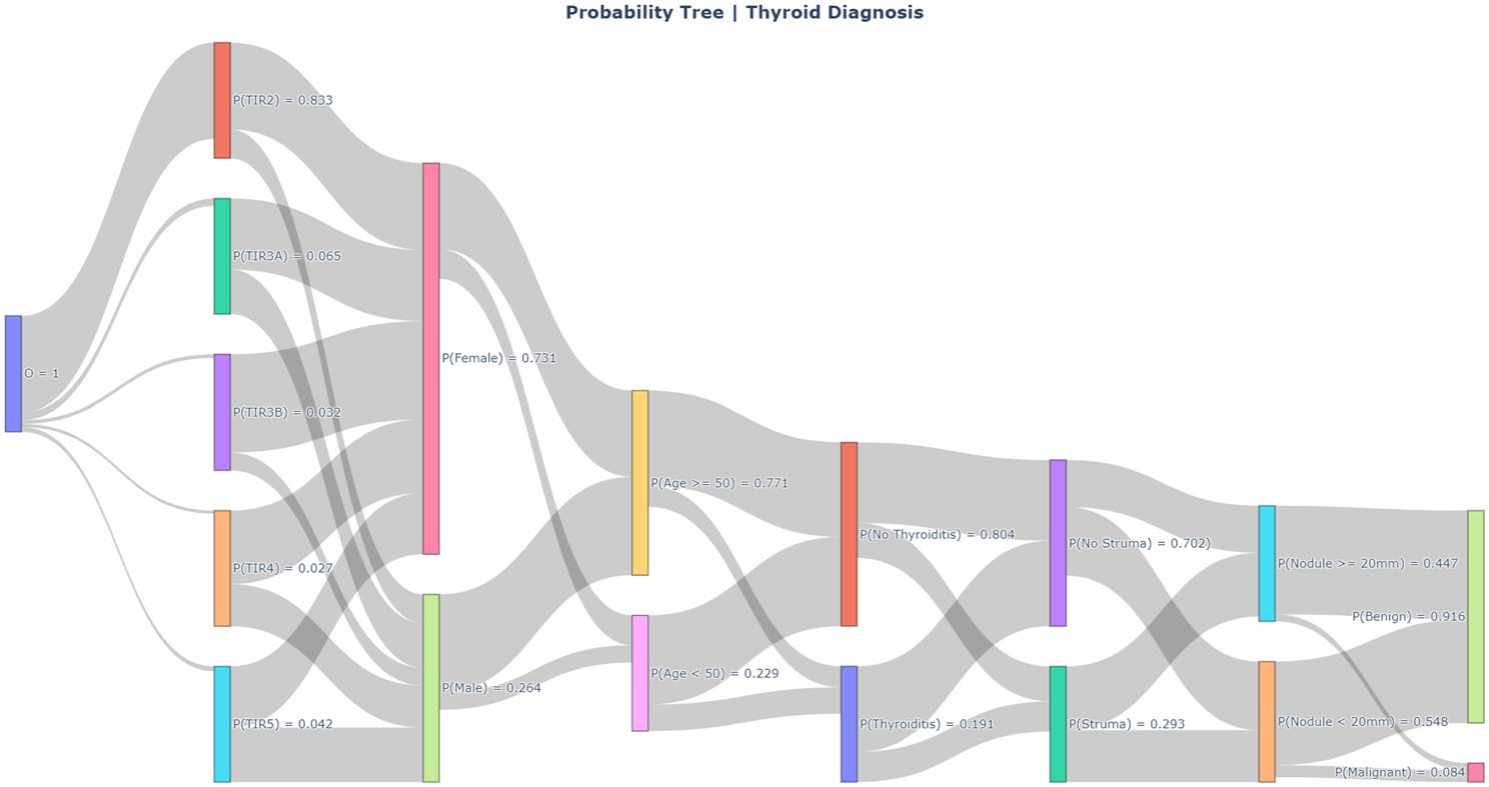
PT developed for the classification of thyroid nodules. Each arc represents the transition probability from one variable to the next, the size of the arcs are representative of the corresponding transition probabilities. The PT has been constructed based on the thyroid nodule data set. As a path is followed along the tree, it will end up in benign or malignant leaf nodes. The majority class in the leaf nodes will be the final prediction (in the case ‘threshold $= 0.5$’ is used)

**Fig. 4:**
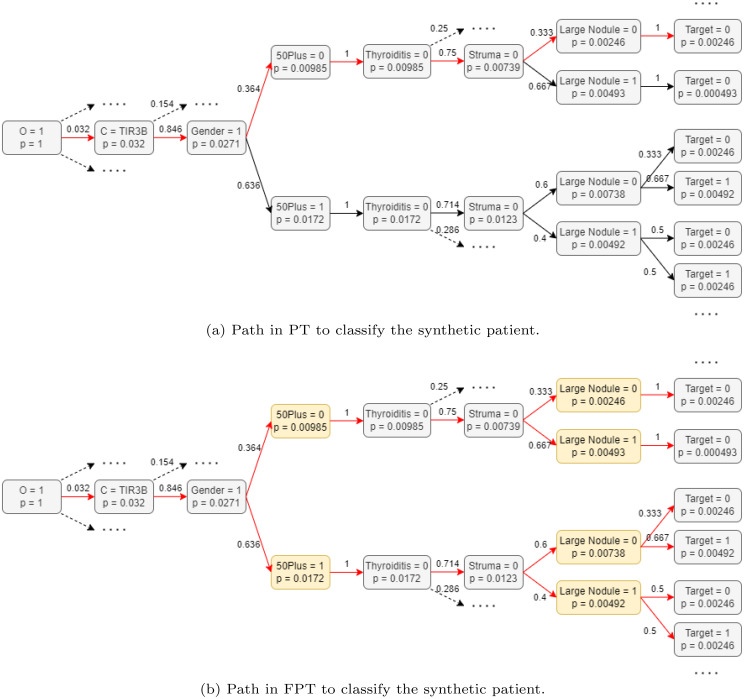
Results of classifying the same patient using PT vs. FPT. The grey nodes denote crisp variables, where yellow boxes denote fuzzy variables. The path that is taken in the tree to classify the synthetic patient is highlighted by the red arrows. In (**b**) FPT, all arrows are red as all ‘paths’ are considered to some degree for the final classification

A schematisation of the probabilistic tree created for this case study is visualised in Fig. 3. It shows the possible values for all variables in the model, the arcs represent the conditional probabilities for the entire data set. This figure does not show every possible total realisation as a separate path in the probability tree, as we can see in the example handled in Fig. 4. Visualising the complete tree will blow up the image, due to the large number of paths. The tree as presented in Fig. 3 will – on average – have 2.5 data points in each total realisation up to a leaf (a total realisation is a path from root the leaf). Although in reality, some patient profiles are more common and the data points tend to clump together while others may not even occur at all. The average number of data points per total realisation is determined by calculating the possible number of combinations of feature values and dividing the total number of data points by this number ($\frac{401}{5 \cdot 2^{5}} = 2.51$).

#### Demo: predicting using the FPT

A demonstration is presented in order to clarify the decision making process of the proposed FPT method and compare it to the traditional PT. We introduce a synthetic patient with the feature values as presented in Table 2. The process of classifying the synthetic patient based on PT and FPT is visualised in Fig. 4a and Fig. 4b, respectively. These figures show only the fraction of the tree that is relevant for the classification of this synthetic patient. The red arrows in indicate the path taken in the tree to make the classification of the synthetic patient. The probabilities on the arcs represent the transition probabilities to go from one node to a following node. The yellow nodes in the fuzzy tree indicate the nodes that correspond to one of the fuzzy variables.

**Table 2:** The synthetic patient used for the demonstration of FPT

Feature	Value
Class	TIR3B
Gender	Female
Age	48
Thyroiditis	No
Struma	No
Nodule Dimensions	18 mm

**Fig. 5:**
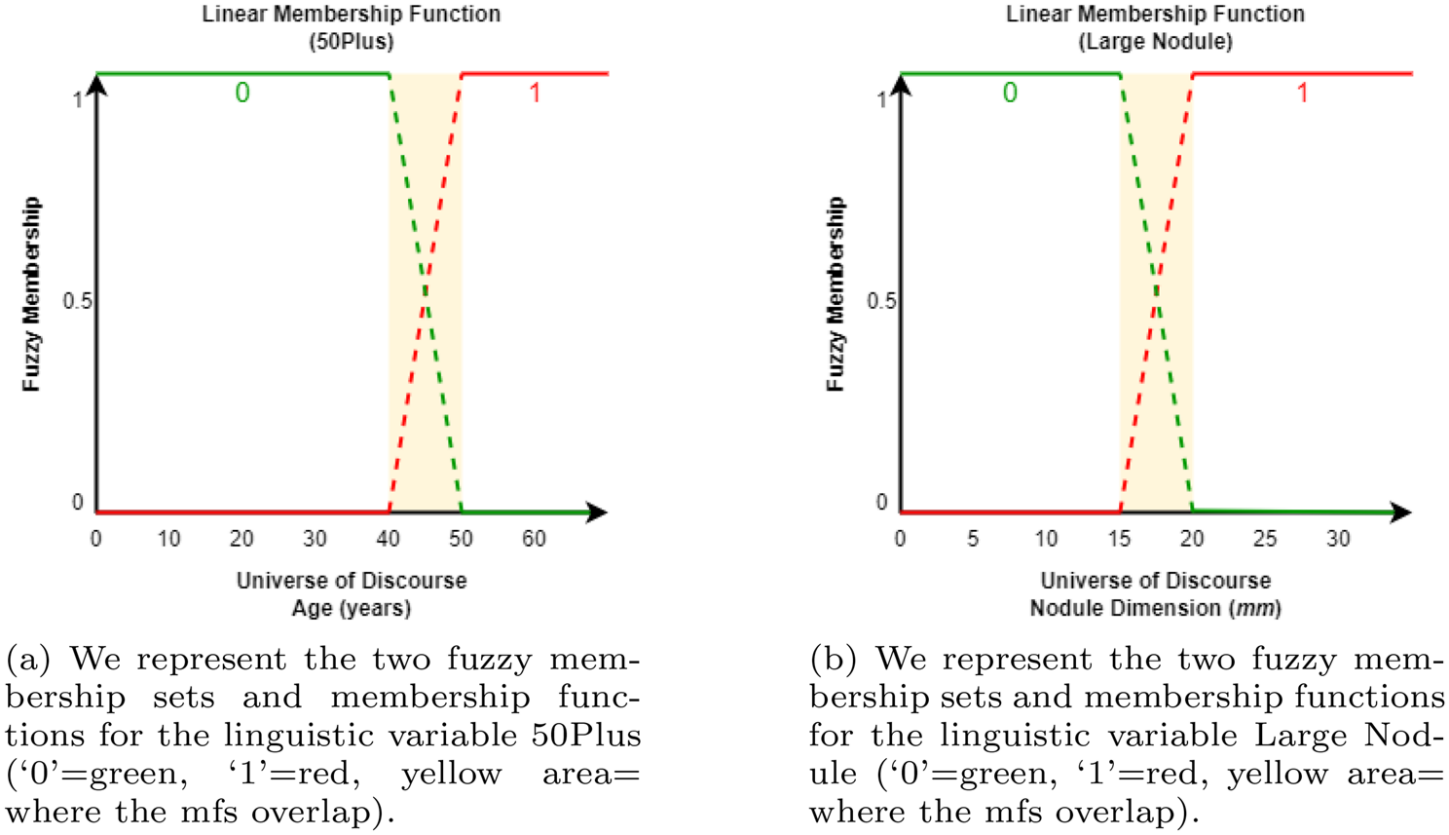
Linear membership functions for the fuzzy variables (*age* and *nodule dimension*)

We transform two features into linguistic features in order for these features to be modelled as fuzzy variables. The feature for the age of a patient is modelled as the linguistic feature ‘50Plus’ and the feature nodule size is modelled as the linguistic feature ‘Large Nodule’. The fuzzy sets are depicted in Fig. 5a and Fig. 5b. In the traditional PT these variables have crisp thresholds. Namely, for the age we make a hard cut-off at the age of 50. A large nodule is defined to be any nodule with dimensions $\geq 20$ millimetre (mm)).

The design of the tree and the transition probabilities do not change from the PT (Fig. 4a) to the FPT (Fig. 4b). However, what is different is when we make a prediction based on the FPT algorithm. In the case that we aim to predict a data point that has a membership degree in the range $0 < mf < 1$, we take the weighted average of multiple observations (i.e., we consider multiple paths in the tree). The outcome is a weighted average, where each path is weighed according to its degree of membership in the fuzzy variable. The PT (Fig. 4a) classifies the synthesised patient to have a benign nodule (‘Target $= 0$’) with a probability of $1$. This prediction follows from simply following the path corresponding to the patient’s information in the tree, which is denoted by the red arrows. The FPT (Fig. 4b) considers all paths as the patient has membership degrees in the range $0 < mf < 1$ for both fuzzy variables. Therefore, all arcs leaving the yellow fuzzy nodes are depicted in red. The FPT classifies the synthesised patient with a benign nodule with a probability of $0.427$.

This demonstrates the important nuances that incorporating fuzziness in features may bring. Considering a patient aged 48 to belong fully to the group of patients that are 50 years or less, leads to a possible loss of important information. As this patient is very close to the age of 50, the patient should be considered accordingly. Similarly, the FPT considers the feature ‘nodule dimensions’ to be a fuzzy variable. Whereas in the crisp PT, a large nodule is defined as any nodule with dimensions larger than or equal to 20 mm. The FPT implements a linear membership function, herein a nodule with dimensions of 18 mm belongs to the class of ‘large nodules’ to a (membership) degree of $0.80$. The written out calculations, showing the integration of the membership values, to obtain the classification for the synthesised patient as made by the FPT is provided in Eq. 2.

2 $$\begin{aligned} \text{Fuzzy prediction of} & \text{synthetic patient} = \\ & 0.2 \cdot \big((0.2 \cdot 1 \cdot 0) + (0.8 \cdot 1 \cdot 0)\big) + \\ & 0.8 \cdot \big((0.2 \cdot 0.333 \cdot 0) + (0.2 \cdot 0.667 \cdot 1) + \\ & (0.8 \cdot 0.5 \cdot 0) + (0.8 \cdot 0.5 \cdot 1)\big) = 0.427.\end{aligned}$$

### A chronic kidney disease case study

Chronic Kidney Disease (CKD) is a condition in which the kidneys are damaged and cannot filter blood the way that they should. Because of this, excess fluid and waste from blood remain in the body and may cause other health problems, such as heart disease and stroke. Prevalence and incidence of CKD have almost doubled in the past two decades [27]. We aim to predict whether patients will progress to End Stage Renal Disease (ESRD), which is the most advanced and harmful stage of kidney disease. In this stage, patients often need dialysis and/or a kidney transplant. Dialysis is a procedure to remove waste products and excess fluid from the blood when the kidneys stop working properly. Thus, it is a therapy that replaces the normal blood-filtering function performed by the kidneys. Determining the probability of kidney failure may be useful for the communication between the clinicians and patients, furthermore, it will be leading in the triage and management of nephrology referrals and the timing of dialysis and kidney transplants. Reliable and interpretable prediction tools are needed to identify patients with CKD that are at greater risk of developing towards ESRD. The progression is often hard to predict, as the disease does not progress in the same rate for all patients. Moreover, it is said that a substantial portion of patients suffering from CKD do not follow a predictable pattern of disease progression.

#### The kidney disease data set

This study includes a data set that is the result of a multi-centre prospective study pooling data from 46 established hospitals and clinics located in the EU. The studies have been conducted for a total of 20 years, starting in 1995 and continuing up to 31 December 2015 [28]. The data set is of tabular form and contains the records of 3.278 patients, each consisting of 131 different features. Table 1 and Fig. 3 in Supplementary File presents an overview of the relevant features and their types for this case study. From the 3.278 records, a total of 674 have been excluded. A number of 236 duplicate patients have been removed, as well as 242 patients that had Glomerular Filtration Rate (GFR) scores that are no indication for CKD. Furthermore, 202 patient files have been excluded due to missing information about the levels of potassium (kalium) and/or protein present in the urine. This leads to a final data set containing the records of 2.599 patients with CKD. For creating, training and testing the model, only complete patient profiles have been used. However, the proposed methods can be very useful in making predictions to support the clinical decision processes for patients with missing information. The FPT algorithm can be used to create counterfactual statements to test out different scenarios (i.e., different possible values for the specific variable) and evaluate these scenarios for its risks, given the patient information that we know for certain. All study participants have signed an informed consent.

A schematization of the probability tree is presented in Fig. 1 of Supplementary File, and consists of features that contain general patient information (age, diabetes), features based on blood tests values (serum creatinine, anemia (hemoglobin), GFR), and features based on urine testing values (proteinuria, phosphate). On average, the tree will have about five data points in each total realization. However, some patient ‘profiles’ will be more common than others. This number is determined by dividing the total number of patients by the number of possible combinations of feature values: $\frac{2599}{4 \cdot 2^{7}} = 5.08$.

Fuzzy sets are created to represent the variables: serum creatinine, anemia, proteinuria, hyperkalemia, phosphate and the age. The aim of creating these fuzzy sets is to create a gradual boundary between the positive and negative class. Each variable is represented using two fuzzy sets, all of which are visualized in Fig. 3 of Supplementary File.

## Results

In this section we apply our knowledge-based PT and FPT for the analysis of the two clinical data sets. The experimental results that have been achieved by our proposed FPT method are presented, alongside results for two other interpretable benchmark models, namely, Logistic Regression and the Decision Trees.

We implement the method of bootstrapping to create multiple data sets based on resampling with replacement. On forehand, the data set is split into train and test partitions that are assured to be representative of the data set with the use of stratification. The performance metrics in terms of accuracy, specificity, sensitivity, and precision along with corresponding confidence intervals (95%) are documented. The predictions are made using a default threshold, which is set at a probability of 0.50. The predicted probability is compared to this threshold, when it is lower than the threshold the predicted classification will be the negative class, when it is higher or equal to the threshold it will be classified as the positive class. This threshold can be adjusted according to the situation. For example, it may be increased in times of low capacity when only the most urgent cases can be assessed, i.e., in situations where prioritisation is required.

### Results: thyroid nodule case

The performance of the PT and FPT for the thyroid nodule case are presented in Table 3, similar results are observed for both approaches. This is due to the similarity of the two models: there are only a few variables that are represented as fuzzy in the FPT and are considered using crisp thresholds in the PT. Furthermore, the models base their predictions on the same tree, the difference is in the handling of the fuzzy variables (age and nodule dimensions). Nevertheless, the FPT slightly outperforms the PT across all performance metrics. The biggest difference is in the sensitivity (FPT scores 1% point higher), which is a key performance metric in a medical applications for it associated to the ability to correctly identify patients affected by a disease. Furthermore, the increment in the lower bound of the CI for the precision of the FPT indicates that it performs more steadily in predicting the disease. The improvement of performance, although slight, suggests that modeling variables using fuzzy sets can improve the capability of FPT of capturing the nuances of a medical scenario.

**Table 3:** Performance of PT, FPT, LR and DT in thyroid nodule case study on accuracy, specificity, sensitivity and precision (95% confidence intervals). Based on 1000 bootstrapped data sets. Threshold $= 0.50$. LR: no penalty, LBFGS solver

	Accuracy (95% CI)	Specificity (95% CI)	Sensitivity (95% CI)	Precision (95% CI)
PT	96.8% [93.1–99]	98.2% [94.6–100]	82.2% [55.6–100]	84.1% [58.3–100]
FPT	96.9% [94.1–99]	98.3% [95.7–100]	83.2% [55.6–100]	84.2% [63.6–100]
LR	97.1% [94.1–100]	98.5% [95.7–100]	82.7% [55.6–100]	85.8% [66.7–100]
DT	96.3% [93.1–99]	98% [94.6–100]	79.8% [55.6–100]	81.5% [57.1–100]

We compared the performance achieved by the PT and FPT to two benchmark models that are also inherently interpretable, namely LR and DT (see Table 3, with corresponding 95% confidence intervals). Both the LR and the DT are able to handle continuous predictor variables, therefore, the original variables are used instead of the linguistic variables that have been introduced for the age and dimensions (‘50Plus’ and ‘Large Nodule’). The rest of the variables are either categorical or binary, and thus, are not changed.

When comparing the performance of the PT and FPT to the performances of the LR and DT (Table 3), both FPT and LR stand out. The LR performs best in terms of accuracy, specificity, and precision. The lower performance in terms of sensitivity indicates that the LR model is more careful in predicting a patient to have the disease, and therefore, being more generous in labeling patients as benign, which is the majority class. If this is indeed the motivation, we can argue that the FPT is in fact the most useful model. However, the performances of all models are quite comparable and they struggle in correctly classifying the same set of data points. Namely the data points that represent patients that are in the TIR3A or TIR3B class (classes based on the biopsy). As there are very few data points present in the data set that have a class TIR3A (2 data points) or TIR3B (5 data points) and end up having a malignant nodule, it is not surprising that the models generally struggle with classifying these data points.

### Results: chronic kidney disease case

In this section we apply our knowledge-based PT and FPT models to make predictions for the CKD case. The results obtained by the PT and FPT models should differ more than in the previous case study. This is the result of a larger number of fuzzy variables present in the model, making the PT and FPT models less similar to each other. This will help us in understanding more about the differences in performance of the PT/FPT.

The performances for the PT and FPT in their predictions for the progression of CKD is presented in Table 4. Comparing the performance based on the reported metrics presented shows that the main difference between the two models lies in the sensitivity scores, where the FPT outperforms the PT by 5.5%. The overall accuracy is slightly improved for the FPT compared to the PT. However, the PT demonstrates slightly better performance for the specificity metric. As regards to the precision, the two models achieve very comparable scores (difference of 0.2% points in favour of the PT). This indicates that the FPTs increase in sensitivity is not achieved simply by the model being more generous with predicting the positive class. Therefore, the results indicate that the FPT is better able to identify the CKD cases that progress to ESRD in the following two year period (the positive class) when compared to the traditional PT. However, it must be noted that both models obtain performance that is generally perceived to be low. The sensitivity and precision scores indicate that the models fall short on predicting the CKD cases that progress to ESRD. Nonetheless, the novel FPT method proves to be an improvement over the traditional PT.

**Table 4:** PT, FPT, LR and DT results on accuracy, specificity, sensitivity and precision (95% confidence intervals (CIs) in CKD case study). Based on 250 bootstrapped data sets. Thresholds used $= 0.50$

	Accuracy (95% CI)	Specificity (95% CI)	Sensitivity (95% CI)	Precision (95% CI)
PT	76.9% [73.7–80]	84.1% [78.4–89.2]	56.7% [47.6–66.4]	56.1% [49.8–63.2]
FPT	77.2% [73.8–80.2]	82.5% [77.3–87.3]	62.2% [52.9–72.3]	55.9% [50–62.2]
LR	82.7% [80.6–84.8]	93.5% [91.5–95.6]	52.2% [45.3–59.4]	74.1% [67.9–80.8]
DT	74.6% [71.7–77.2]	82.2% [78.3–85.6]	53.1% [44.7–61.1]	51.5% [46.2–56.8]

The performances are compared to two interpretable benchmark models: LR and DT. The models are given the same combination of features as the (F)PT models. However, LR and DT are both capable of handling continuous predictor variables, therefore, the continuous values of the variables are used. As opposed to the (fuzzy) binary variables that were created for the PT and FPT, that divided the data points into two divisions using splits. The performances for the two benchmark models, based on the four performance metrics, are presented in the second half of Table 4. Comparing the performance metrics of all models a similar pattern (as in the previous case study) can be observed. The LR is superior in terms of accuracy, specificity, and precision. However, the FPT achieves the highest score in sensitivity. The LR is most accurate in predicting the majority class, however, it is more careful in predicting the positive class (i.e., the minority) and, thus, misses many cases of the disease while scoring higher on the accuracy metric. The FPT is better capable in identifying the positive class, however, its precision in doing so is substantially lower than what is obtained by the LR. Again, it must be noted that all models are limited in their performance, and may not display the performance desired to assist in a clinical environment.

### Interpretability analysis

Although the need for interpretability in machine learning models has been established, there is no agreed upon definition of what constitutes interpretability [29, 30].

In this work, we adopt the dichotomy of Linardatos et al. [29]:

- Interpretability: AI systems that are intrinsically understandable by users. Interpretability is a global property of the AI system;
- Explainability: *ex post* analysis of the machine learning model, in order to understand the motivations that led the AI system to a specific prediction. Since the explanation is built with respect to one specific input, it represents a local property.

Similarly, Lipton et al. proposed to define interpretability as a system such that (1) gives insights into how the model works in a way that is understandable to a human, and, (2) reveals potential new knowledge. This definition embraces PFT and clearly excludes *post hoc* explainability. Given the aforementioned definitions, we can compare the DT, PT, FPT and LR models according to the their concrete interpretability.

DTs and FPTs are both based on rules that are induced from their respective trees. As the depth of a tree increases, the rule itself becomes more and more complicated (the length of each rule will be equal to the depth of the tree, i.e., the number of nodes that are in the full realization). Differently from DTs, though, FPTs have an important advantage: the output produced by the same rule is presented in the form of probability instead of a final predicted class/outcome.

LRs also models the probability of an event to occur. In this case, the LR maximizes the log odds (natural logarithm of the odds) by optimizing the regression coefficients, which makes it difficult to directly interpret and understand the model. As a result, FPTs (and DTs) are more inherently comprehensible for humans, as their outputs can be translated to natural language in the form of rules. Although the inner workings of an LR may not be as inherently comprehensible, however, the outputs it presents are indeed interpretable and it is capable of presenting clear feature weights, indicating the effect on the target variable after a change in a certain feature value. Although these elements could be “interpreted” by a data scientist or software engineering, they may be challenging for any scientist coming from a different discipline and lacking a formal training about mathematical models, data analysis and machine learning. This circumstance implies that actual *understandability* of LR models is not really achieved [31] without previous background knowledge. On the contrary, in the case of DTs and PTs, the amount of previous knowledge and expertise that is necessary to understand the functioning of the system and its prediction is much more limited, because the user can answer the questions corresponding to nodes, follow the edges along the tree, and obtain a final probability prediction. The fuzziness in FPTs provides a sound mathematical framework to accommodate the inherent uncertainty of some variables, at the cost of a slightly higher cognitive load due to the necessity to consider multiple output scenarios.

### Potential of the FPT method (counterfactuals)

It is important to note that the full potential of the fuzzy probability tree may be yet to be discovered. As probability trees are causal models that use nodes to represent potential states of a process, therefore, the arcs between nodes indicate the probabilistic transition, but preferably also the causal dependency between two states of the process [16]. For the two implementations discussed within this research, the trees did not reflect clear processes wherein every node represents an actual state of the process that is a measurable moment in time. Consequently, it is hard to prove any causality between nodes. Possibly, the full potential of (F)PT will be used when there is a clear temporal aspect in the process that the tree aims to model, and the nodes represent actual states of a process.

Furthermore, an important power of (F)PTs, lies in its ability to consider counterfactual statements. Counterfactuals allow for the testing of alternate realities, meaning that a doctor may test hypotheses that represent slight alterations to the factual situation. An example question that may be answered with the help of counterfactual statements in the CKD case study is: “*Given a patient that developed kidney failure and did not take any medication. What would be the probability of this patient developing kidney failure if he/she had in fact taken RASI medication?*”. However, careful consultation with clinicians is necessary in creating such counterfactual statements, as RASI medication may induce hyperkalemia, which is again a potentially deadly condition. Nonetheless, this allows clinicians to reason about how the situation may have unfolded had other courses of action been taken. This ability to think in counterfactual statements is natural to humans and it is what sets out human intelligence from other animals. When implementing (F)PTs, this human way of reasoning can be leveraged in AI methods that are also able to consider large amounts of data.

### A tool for clinicians

Clinicians usually lack a proper background in ML, and have a limited knowledge of probability theory and statistics. In order to create an effective tool for clinicians to assist complex decision making, the information must be well conveyed and *intelligible* [32], taking into consideration the background knowledge and, possibly, cultural aspects [31]. To this aim, we developed an interactive tool to obtain relevant and naturally understandable statements. Such statements are written in the form of probabilities, obtained from the underlying probabilistic decision tree that gets generated based on past patient records.

Differently from conventional ML approaches, our model does not give a “simple” binary answer (e.g., benign or malignant module) but presents informative probabilities and presents the case of a specific patient, based on information introduced by the clinician, providing the expert with a clear motivation on the patient’s risk of malignancy. Moreover, the clinician can interact with the tool and change, or filter out, some specific conditions to see the impact to probabilities in real-time. This allows the clinician to produce counterfactual statements, and see what the probability for malignancy would be in an alternate reality. This possibility can be very useful in testing the effects of certain interventions on the target variable (where applicable). For instance, counterfactual statements could be leveraged to identify the effects of RASI drugs on the progression of CKD. RASI is medication that reduces the future rate of loss of GFR (key indicator of kidney functioning), however, doctors are careful in prescribing it because RASI medication may induce hyperkalemia, which is again a potentially deadly condition. Testing alternate scenario hypotheses such as “*Given that this patient has progressed to ESRD, if this patient had taken RASI, what would then be the probability of progressing to ESRD?*” can be very valuable. Such statements allow for the integration of counterfactual reasoning, human-like reasoning in terms of the fuzziness in variables and large amounts of data.

Our tool, consisting in a Graphical User Interface (GUI) wrapping the PFT models, was created in the Python 3 programming language and depends on PyQT5 [33], Pandas [34], NumPy [35], Simpful [36], and the probability tree algorithms as proposed by [16]. When launching the GUI, all relevant probabilistic trees and fuzzy sets are created based on a csv file from which it reads the data. When new entries are added to the csv file, these will be considered when creating the tree the next time the tool is launched. Access to the scripts for the FPT algorithm and tool is available upon request.

## Discussion

The future of medicine seem to lie in interactive intelligent systems where human and AI can interact. Thanks to machine learning methods based on fuzzy logic, doctors can leverage techniques that are able to process large quantities of data and generate a diagnosis for decision support systems while also providing an estimate of the degree of uncertainty. It is emerging that medical practice everywhere should be leveraging on sharing information and data in order to improve general healthcare. Implementing such systems will reduce the variability of healthcare among doctors, hospitals and countries.

Although Deep Learning has achieved expert-level expertise in many medical areas [37–41], it is nevertheless essential to aim for interpretability in these systems. We believe that interpretable AI is the most a cautious and promising approach for the development of medical products, with respect to the less strict concept of explainability. Several regulations imposed by the EU enforce that interpretability is part of the contract. The GDPR states that: “*... individuals should not be subject to a decision that is based solely on automated processing (such as algorithms) and that is legally binding or which significantly affects them.*” [7]. Furthermore, regulations like the AI Act [8] aim to specifically regulate the development and use of AI in safety-critical systems (such as the field of medicine and healthcare). Any clinical decision support system is subject to a strict set of requirements; in particular, transparency and interpretability. Interpretable AI systems also offer another interesting possibility, which is the direct verification of existing biases. As a matter of fact, by inspecting the effect of branching to the predicted output, one can discover unfair correlations between some variables and the response of the system. Since the FPT is grounded on training data, one can investigate whether the dataset is biased or there is an existing bias in society, which percolated into the AI system. If the bias is caused by a very limited number of samples (a circumstance that can be verified very easily using FPTs), then the solution would be to collect some additional samples and repeat the training. Fairness due to biased or poorly collected datasets is a sensitive topic, which could benefit from Interpretable AI systems like FPTs [42].

The results imply that IAI that mimics human reasoning has a fair shot at supporting clinicians in decision making. Allowing the fuzzy, vague and ambiguous medical variables to have fuzzy boundaries will only improve the model and make it even more human-like, while being able to handle large quantities of data. However, more research is needed to identify for which cases the methods work best. As the proposed methods did not achieve consistent results in the two presented case studies. However, the method is successful in it being inherently interpretable, outperforming traditional probability trees, and a closer resemblance to human reasoning which allows it to consider counterfactual statements in combination with fuzzy variables. In the context of nephrology, and CKD in particular, the current prediction models are based on Cox proportional hazard models. These models often provide estimates on the future risk of future outcomes for each unit increase of a continuous variable or a specific risk category. The improvement of interpretable FPT may be useful to reach a better prediction of future risk (for ESRD as well as for cardiovascular and mortality events) regardless of a specific cut-off. Such a opportunity my overcome the limitation of generate risk categories that can be approximately, and this is particularly true, for instance, for serum bicarbonate and systolic blood pressure or proteinuria [43].

## Conclusion

A novel interpretable decision making method, based on probabilistic trees and fuzzy set theory, was proposed in this paper. The framework, named FPT, exploits the benefits of incorporating fuzzy sets in a probabilistic tree model to support in clinical environments where (some) variables are vague, uncertain or ambiguous. A FPT is an inherently interpretable decision making method, that can be inspected *ex ante* by domain experts, making it human-understandable by design and not requiring *post hoc* explainability; in order to maximize the effectiveness for users, we also developed a GUI around the modeling approach.

We tested and validated the FPT on two distinct clinical case studies: the first was based on patients with thyroid nodules that needed to be classified as benign or malignant; the second case was based on a dataset of patients suffering from chronic kidney disease, for whom the risk in the following 2-year period to experience kidney failure needed to be assessed. The FPT built with our approach was compared against several common interpretable benchmark models. Overall, the FPT achieved the best score in detecting the patients that suffer from the disease (i.e., the highest sensitivity) in both case studies. This novel interpretable fuzzy probabilistic decision making approach was therefore shown to be a promising addition to the literature on IAI. Incorporating fuzzy variables into the probabilistic model has shown to better reflect the reality of the vagueness that exists in clinical variables.

We remark that, although the FPT presents an improvement over PTs and to some extent also over a logistic regression, the performance obtained may not yet be sufficient to serve as a reliable decision support tool to assist in daily clinical decision making processes. In future work we aim to test our methods on a larger and external cohort, focusing on addressing current limitations and assessing the performance of the FPT to enhance its reliability and clinical applicability. Furthermore, as a future extension of this work, we plan to research the automatic generation of probabilistic trees – combined with feature selection – by means of combined modeling and meta-heuristics optimization [44]. Finally, we plan to extend the FPT to natively support non-binary target variables.

## Electronic supplementary material

Below is the link to the electronic supplementary material.

Supplementary Material 1

## Data Availability

The datasets used and/or analysed during the current study are available from the corresponding author on reasonable request.

## Code availability

The code used in the current study are available from the corresponding author upon request.

## Declarations

### About this supplement

This article has been published as part of BMC Medical informatics and Decision Making, Volume 25 Supplement 3, 2025: IEEE CIBCB 2023 - 20th IEEE Conference on Computational Intelligence in Bioinformatics and Computational Biology. The full contents of the supplement are available at https://bmcmedinformdecismak.biomedcentral.com/articles/supplements/volume-25-supplement-3.
