# Supplementary material for: Assisting clinical diagnosis with interpretable fuzzy probabilistic modelling
Source: BMC Med Inform Decis Mak. 2025 Sep 15;25(Suppl 3):330. doi: 10.1186/s12911-025-03183-5 (PMC12439376; doi:10.1186/s12911-025-03183-5)
Supplement: Supplementary file 1 — Supplementary Material 1 [file 12911_2025_3183_MOESM1_ESM.pdf]

Assisting Clinical Diagnosis with Interpretable Fuzzy Probabilistic Reasoning

Supplementary Material

Giulia Capitoli[1,3], Marco S. Nobile[2,3,6], Emma L. Ambags[2], Vincenzo L'Imperio[4], Michele Provenzano[5], and Pietro Liò[7]

[1]School of Medicine and Surgery, University of Milano-Bicocca, Monza, Italy
[2]Eindhoven University of Technology, Eindhoven, The Netherlands
[3]Bicocca Bioinformatics, Biostatistics and Bioimaging (B4) research center, Milan, Italy
[4]Department of Medicine and Surgery, Pathology, University of Milan-Bicocca, IRCCS Fondazione San Gerardo dei Tintori, Monza, Italy
[5]Nephrology, Dialysis and Renal Transplant Unit, IRCCS—Azienda Ospedaliero-Universitaria di Bologna, Alma Mater Studiorum University of Bologna, 40126 Bologna, Italy
[6]Department of Environmental Sciences, Informatics and Statistics (DAIS), Ca' Foscari University of Venice, Venice, Italy
[7]Department of Computer Science and Technology, University of Cambridge, Cambridge, United Kingdom

1 Membership functions and natural language

Fuzzy sets are mathematical concept that allows for uncertainty and partial membership to a set. Differently from traditional sets, where elements can either belong or not belong to a set, in fuzzy set they can have varying degrees of membership. Such degree of membership for an element $x \in \Omega$, where Ω is the universe of discourse, is represented and formalized with a Membership Function (MF) $\mu(x) \in [0, 1]$. Five examples of membership functions are shown in Figure 1. The MFs shown in the figure are: (a) rect trapezoid (b) triangular (c) trapezoid (d) Gaussian (e) sigmoid.

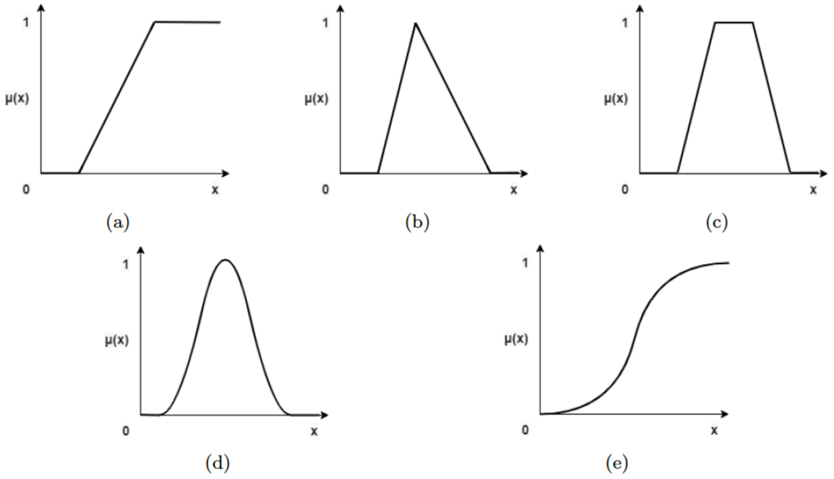

Figure 1: Example of membership functions.

These MFs can be used to model vague and fuzzy concepts. For instance, the body temperature of a patient can be "high". Although a measured temperature greater than or equal to $38°C$ is usually assumed to be high, also a slightly lower temperature should be considered in a similar way, with a reduced membership degree. This is the way humans (and medical doctors in particular) generally think. That line of reasoning is what a MF like Figure 1(a) represents: the curve is 0 for very low values, slowly increases for medium values, and reaches 1 for high values. A similar idea, although based on a smoother approach, is represented by the sigmoidal MF in Figure 1(e): the higher the x value, the higher the membership function.

It is possible that the fuzzy concept to be modelled represents a basic condition, e.g., a basal interval of values in non-pathological conditions. This idea can be modelled using the MFs in Figures 1(c) and 1(d). A MF like 1(b) can be used when the interval to be modelled is not symmetrical.

Linguistic variables can be created using multiple linguistic terms that are typical of medical terminology (e.g., "low", "medium", "high", "a lot", "a few", "highly expressed"), where each term is modelled using a dedicated MF, similar to those in Figure 1.

2 Chronic kidney disease case study

Table 1: Explanation of relevant features.

**Description**	**Type**
Date of birth	Ordinal
Gender	Binary
BMI	Quantitative
Diabetes	Binary
Smoking	Binary
CVD	Binary
Kalium (mmol/l)	Binary
GFR (mg/mmol)	Binary
Stadium of GFR	Ordinal
Serum Creatinine (mg/dL)	Quantitative
Proteinuria (g/24h)	Quantitative
Hemoglobine (g/dl)	Quantitative
Phosphate (mg/dl)	Quantitative
RASI medication	Binary
Date of visits	Ordinal
Death pre-dialysis	Binary
ESRD	Binary

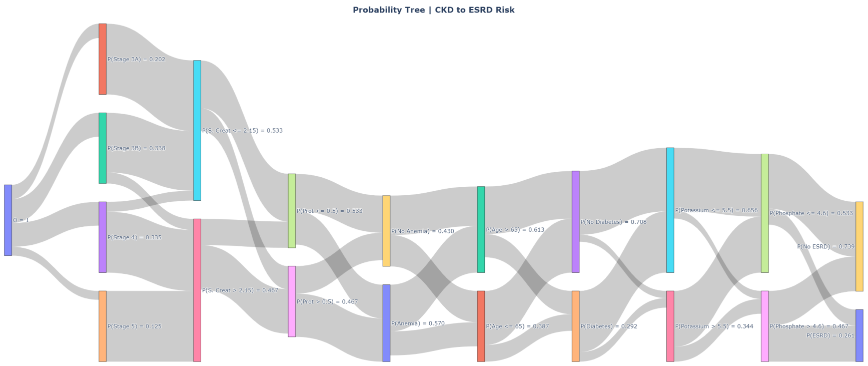

Figure 2: PT developed for predicting the risk of CKD patients progressing to ESRD. Each arc represents the transition probability from one node (variable) to the next, the size of the arcs are representative of the corresponding transition probabilities.

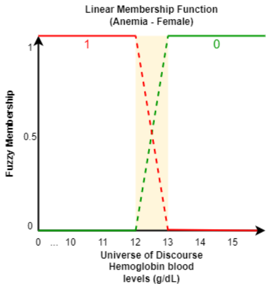

(a) Fuzzy sets for the variable Anemia (females).

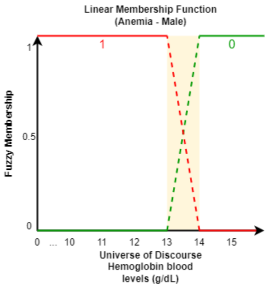

(b) Fuzzy sets for the variable Anemia (males).

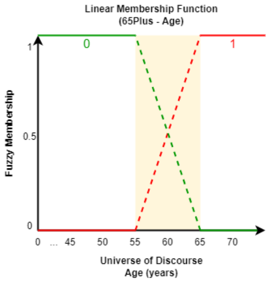

(c) Fuzzy sets for the variable age (65Plus).

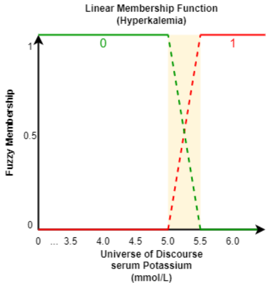

(d) Fuzzy sets for the variable hyperkalemia (serum potassium).

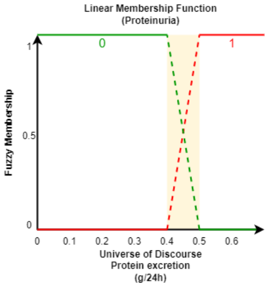

(e) Fuzzy sets for the variable proteinuria.

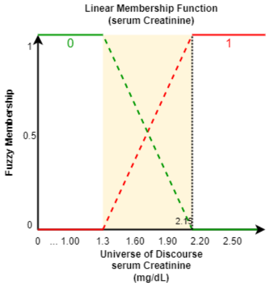

(f) Fuzzy sets for the variable serum creatinine.

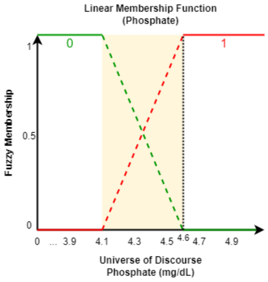

(g) Fuzzy sets for the variable Phosphate.

Figure 3: Fuzzy sets and linear membership functions for the variables: anemia (males and females), age, hyperkalemia, proteinuria, serum creatinine, and phosphate ('0'=green, '1'=red, yellow area= where the sets overlap and thus become fuzzy).
